# Clinical-Microbiological Study of Nontyphoidal Salmonella Infections from Karnataka, India

**DOI:** 10.1155/2024/6620871

**Published:** 2024-07-16

**Authors:** Biranthabail Dhanashree, Shalini Shenoy

**Affiliations:** Department of Microbiology Kasturba Medical College Mangalore Manipal Academy of Higher Education, Manipal, 576104, Karnataka, India

## Abstract

**Background:**

Nontyphoidal salmonella (NTS) are responsible for food-borne gastroenteritis and bacteremia, in immunosuppressed individuals. Antibiotic resistance in NTS is on the rise. This study reports the prevalence and antimicrobial susceptibility of nontyphoidal salmonella in clinical samples. *Study Design*. This is a hospital record-based cross-sectional, retrospective study.

**Methods:**

In our study, inpatient samples such as blood (*n* = 13546), urine (*n* = 11,333), pus (*n* = 1,010), and stool (*n* = 926) samples were cultured to isolate etiological agents in the microbiology department from January 2017 to June 2019. Relevant details such as duration of fever, platelet count, hemoglobin, WBC count, immune status, and mode of treatment were noted from the medical records. Data were analyzed, and continuous variables were expressed as mean and categorical variables as percentages.

**Results:**

A total of 24 NTS serovars were isolated, which included ten isolates from blood, five each from urine and pus, and four from stool samples. Of the NTS, five were *Salmonella enterica* subsp. enterica, three were *S.enterica* serovar Typhimurium, one was *S.enterica* subsp. diarizonae, and 15 *S.enterica* serovars could not be serologically differentiated. All were susceptible to ampicillin, chloramphenicol, and cefuroxime. However, 16.6% of NTS was resistant to ceftriaxone, 4% to cotrimoxazole, 58% to ciprofloxacin, and 75% to nalidixic acid.

**Conclusions:**

A low prevalence of NTS responsible for invasive infections is seen in this part of the country. Few isolates were resistant to more than one antibiotic. A higher rate of resistance to ceftriaxone is the cause of concern. Awareness of the distribution of NTS serogroups is of epidemiological and public health significance.

## 1. Background

Nontyphoidal salmonella (NTS) serovars infect a wide range of hosts. They are responsible for self-limiting diarrheal disease and its carriage in immunocompetent humans [1]. NTS are crucial pathogens that are responsible for food-borne gastroenteritis and bacteremia in immunocompromised patients. They are liable for a substantial global burden of morbidity and mortality [2]. Invasive nontyphoidal salmonellosis is increasingly seen among immunocompromised or malnourished individuals, especially children having HIV infections. In resource-limited countries, the case fatality rate is remarkably high due to an increase in resistance to drugs used for treatment such as quinolones, third-generation cephalosporins, ampicillin, and cotrimoxazole [2, 3].

NTS spread beyond the gastrointestinal mucosa to infect sterile sites, such as the bloodstream, bone, joints, and meninges [4]. A study from Michigan, United States, reports the increasing resistance of NTS to multiple antibiotics and its association with longer stay in the hospitals for treatment [5]. Therefore, preliminary studies on NTS must include its antibiotic susceptibility pattern along with its prevalence.

Mangalore, which is endemic for enteric fever and microbiology laboratory at KMC Hospital, receives clinical samples from patients with acute febrile illness for the diagnosis of etiological agents. Hence, the present study is performed to know the proportion of NTS and its antimicrobial susceptibility pattern in this region.

## 2. Methods

This is a cross-sectional, retrospective hospital record-based study which includes patient data collected from January 2017 to June 2019 from an 850 bedded tertiary care hospital. Patients admitted to KMCH, Mangalore, were included after obtaining clearance from the Institutional Ethics Committee (Ref No.: IEC KMC MLR 09-16/208). A total of 26,815 clinical samples were cultured in the KMC microbiology laboratory to identify the etiological agents of the disease. These samples included 13,546 (50.51%) blood samples, 11,333 (42.26%) urine samples, 1,010 (3.76%) pus samples, and 926 (3.45%) stool samples. Since it is a retrospective study, we were unable to obtain the clinical details of outpatients. Hence, all outpatient samples were excluded from the study.

Patient samples were cultured using standard microbiological methods. The identification of the isolates and antibiotic sensitivity was performed using VITEK 2 system. Polyvalent (A–G) and monovalent antisera were used for serotyping of *Salmonella* spp. It was purchased from the Central Research Institute, Kasauli, India. Clinical details of patients from whom NTS was isolated were collected from the patient's case records. The details included were the duration of fever, platelet count, hemoglobin, WBC count, ESR, results of liver and renal function tests, and mode of treatment. The clinical, microbiological, and demographic data of the patients were analyzed. The Clinical and Laboratory Standards Institute (CLSI) guidelines were used to interpret antibiotic susceptibility results [6].

### 2.1. Analysis of the Results

We used SPSS software (Statistical Package for the Social Sciences; IBM Corporation, NY, USA), version 20.0 for the analysis of the results. The prevalence of NTS was calculated and stated as percentage in relation to the total number of samples tested. Data were expressed as mean ± standard deviation, percentages, and proportions.

## 3. Results

**Figure 1:**
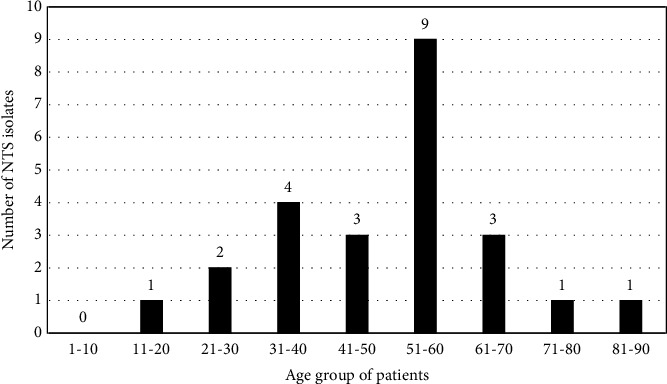
Demographic characteristics of the study population.

From January 2017 to June 2019, a total of 26,815 clinical samples were cultured in the microbiology laboratory to identify the etiological agents of the disease. Of which 13,546 (50.51%) were blood samples, 11333 (42.26%) urine samples, 1010 (3.76%) pus samples, and 926 (3.45%) were stool samples. The mean age of male patients infected with NTS was 53.10, and that of females was 58.20. The male-to-female ratio was 3.8 : 1. The demographic data of the patients infected with NTS are shown in Figure 1.

**Figure 2:**
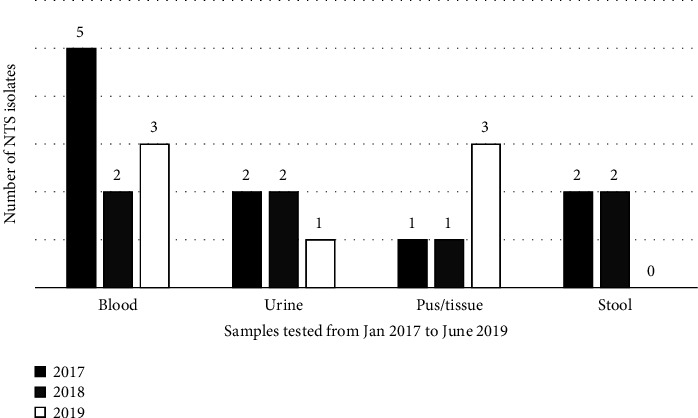
Year wise distribution of nontyphoidal Salmonella serovars in clinical samples.

Among the 26815 samples processed, 24 (0.08%) showed the growth of nontyphoidal *Salmonella* serovars. Among these NTS, ten isolates were from blood, five each from urine and pus, and four from stool samples. The sample-wise and year-wise distribution of various *Salmonella* serovars during the study period is shown in Figure 2.

The maximum number of NTS was isolated from patients in the age group of 51-60 years. All 24 patients (5 females and 19 males) who were positive for the NTS culture had a fever for 5 to 8 days, with headache and myalgia. Four patients had diarrhea in addition to these symptoms. Only three patients had elevated levels of transaminases, alkaline phosphatases, serum bilirubin, creatinine, and blood urea. The blood pictures of the patients showed thrombocytopenia ranging from 7000 to 1, 25000 cells/mm^3^, and leukocytosis (10,100 to 23,000 cells/mm^3^) raised ESR (30 to 70 mm/1st hr.).

Depending on the site of salmonella isolation and the clinical signs of the patients, they were categorized into three groups. (i) Patients with intestinal salmonellosis (*n* = 4): those with diarrhea and positive fecal culture. (ii) Patients with extra intestinal salmonella infections (*n* = 10): those in whom salmonella was isolated from samples other than blood and stool. (iii) Patients with bacteremia (*n* = 10): those with a positive blood culture without other focus of infection.

Twenty-one patients had diabetes with elevated blood glucose levels ranging from 200 to 350 mg/dl. Among these diabetes patients, 10 (nine in the age group 51 to 60 years and one in 61 to 70 years) had bacteremia and positive blood culture with no other focus of infection. Seven had an extraintestinal infection, as salmonella was isolated from urine (*n* = 5) and pus (*n* = 2) samples. Four patients had intestinal salmonellosis with stool culture showing growth of salmonella.

Among the remaining three patients (age group 41-50 years), one had carcinoma of the lung, the other had adenocarcinoma of the liver, and the third had acute suppurated lymphadenitis whose pus cultures were positive and blood cultures were negative. All these 24 patients were treated with ceftriaxone in addition to supportive treatment. Twenty-one patients survived, and three died. Additional investigations were carried out in all these 24 patients with pyrexia of unknown origin to arrive at a final diagnosis. Their peripheral smears were negative for malaria and filariasis. The serology for human immunodeficiency virus (HIV), hepatitis B, C, and E virus, was negative. Their Widal and Weil-Felix tests were also negative. Different *Salmonella* serovars were isolated, and their susceptibility pattern is shown in Table 1.

VITEK 2 system was used to identify all suspected salmonella isolates. Confirmed with agglutination test using polyvalent (A–G) and monovalent antisera. Among the 24 NTS isolates, five were *Salmonella enterica* subsp. enterica (4 stool and one urine isolate), three were *S. enterica* serovar Typhimurium (blood isolates), and one was *S. enterica* subsp. diarizonae from pus. Fifteen isolates were biochemically identified by the VITEK 2 system as salmonella group and agglutinated with antisera A–G specific to the salmonella group. These isolates could not be serotyped at our laboratory. They became nonviable during transport to the reference center and hence could not be further characterized.

## 4. Discussion

NTS serovars, being zoonotic agents, have an extensive range of animal reservoirs. Infections due to NTS include, but are not limited to, mild diarrheal disease and severe systemic infections [1]. Diarrhea due to NTS is a self-limiting disease and does not require antimicrobial treatment in immunocompetent individuals. However, antibiotic treatment is necessary for the management of NTS-infected patients who are at extremes of age, immunocompromised, and with systemic invasive diseases. In our study population, 24 NTS-infected patients were treated with antibiotics as they were immunocompromised and had a fever.

For the diagnosis of nontyphoidal invasive salmonellosis, reliable rapid diagnostic tests are not available, and culture is the gold standard, though slow and insensitive [2, 4]. In our study, we have isolated NTS from blood, pus, urine, and stool samples (Figure 2). However, the isolation rate was 0.7 per 1000 in blood, 0.4 per 1000 in urine, 5 per 1000 in pus, and 4 per 1000 in stool samples. This rate of isolation is much lower when compared to the isolation rate reported in Indian and Asian studies, which ranges from 6 to 8 per 1000 cases [7–10]. Moreover, most of these Indian studies report the isolation of NTS from stool samples. A study from Hyderabad, India, reports the isolation of NTS from blood culture [11]. In our study, a greater number of NTS were isolated from blood, urine, and pus samples when compared to stool samples. This is an indication that invasive NTS (iNTS) is more common in Mangalore than NTS causing gastroenteritis. Studies from Vietnam and African countries and from Hyderabad, India [9, 11, 12], report the isolation of iNTS from human clinical samples, which is consistent with the findings of our study. However, the burden of iNTS in India is not yet clear [10]. In our area, different *Salmonella* serovars were associated with infections in immunocompromised individuals. Few of the NTS isolates could not be serotyped which is one of the limitations of our study. Previous studies in India and other parts of the world showed a difference in the distribution of different *Salmonella* serovars mostly in the gastrointestinal tract [7–10]. The present study reports the isolation of the iNTS in immunocompromised patients.

The clinical signs and symptoms, and the hematological parameters noted in the present study are similar to those reported in the previous study [11]. It has been reported that iNTS infections in Southeast Asia are limited to immunocompromised and cancer patients compared to those in Africa, where it is seen in HIV-infected individuals. Even in other countries such as Vietnam, although HIV infection is low, iNTS is greater in HIV-infected patients and adults using intravenous drugs [12, 13]. However, a study from Taiwan reports the risk factor for iNTS bacteremia as old age, systemic lupus erythematosus, and immunodeficiencies other than acquired immunodeficiency syndrome [14]. In the present study, iNTS-infected patients were neither drug abusers nor HIV serology positive. On the other hand, 21 of our patients had diabetes, and three were cancer patients. Thus, our results clearly show that diabetic patients, especially in the age group of 51 to 60 years (Figure 1), are more prone to invasive nontyphoidal salmonellosis in this part of the country.

A study from South India showed that NTS isolates were sensitive to all antibiotics except ciprofloxacin and nalidixic acid [7, 11]. In the present study, 16.6% of NTS were resistant to ceftriaxone, 4% to cotrimoxazole, 58% to ciprofloxacin, and 75% to nalidixic acid (Table 1). Therefore, 19 patients were treated with ceftriaxone, and only four were treated with ciprofloxacin from whom drug-sensitive NTS was isolated. The emerging resistance to ceftriaxone in NTS was reported to be 5% in Tamil Nadu, India [15], which is less than the ceftriaxone resistance reported in our study. Karunakar et al. (2014) have reported a variable resistance rate (7.1 to 48.0%) to ciprofloxacin [16]. A recent study from South India reports that 5% of the NTS isolates from stool samples were multidrug resistant [10]. In our study, the highest resistance to ciprofloxacin was found in the salmonella group, which could not be further characterized. Taneja et al. have reported 25% of their isolates as multidrug-resistant, of which 53.4% are extended-spectrum beta-lactamase (ESBL) producers [17]. However, in the present study, we have not detected any ESBL-producing NTS isolates, and multidrug resistance was observed in only two isolates. Thus, it is evident that the antibiogram of NTS varies in various parts of the country.

The overall isolation rate of NTS from clinical samples was found to be 0.1%. However, an earlier study from Tamil Nadu reports a prevalence rate of 1% of NTS from clinical samples [15]. This clearly shows that the prevalence rate varies according to the geographical area. Since ours is a retrospective study, we could not perform a molecular analysis of the serotypes and find the genes responsible for drug resistance. Therefore, more multicentric studies that focus on the prevalence of NTS in clinical samples and their molecular typing are necessary to control the spread of *Salmonella enterica* subsp. enterica.

## 5. Conclusion

The current study shows a low prevalence of NTS in this part of the country, among which the majority were responsible for invasive infections. These infections were associated with diabetics and cancer patients. Few NTS isolates were resistant to more than one antibiotic. The higher resistance rate of ceftriaxone observed in our study is of great concern. Awareness of the distribution of NTS serogroups and continuous monitoring of their antibiotic resistance are necessary to control the spread of drug resistance and prevent the invasive infections caused by *Salmonella enterica* subsp. enterica in immunocompromised individuals.

## Figures and Tables

**Table 1:** Antibiotic susceptibility pattern of nontyphoidal salmonella serovars.

Antibiotic	Standard MIC breakpoints (as per CLSI guidelines) (mg/L)			Salmonella group (*n* = 15)		*Salmonella* enterica (*n* = 05)		*Salmonella* typhimurium (*n* = 03)		*Salmonella* diarizonae (*n* = 01)	
	S	I	R	S	R	S	R	S	R	S	R
Ampicillin	≤8	16	≥32	15	0	5	0	3	0	1	0
Chloramphenicol	≤1	2	≥4	15	0	5	0	3	0	1	0
Ceftriaxone	≤1	2	≥4	11	4	5	0	3	0	1	0
Cotrimoxazole	—	—	≥4/76	15	0	5	0	3	0	0	1
Ciprofloxacin	≤0.06	0.12–0.5	≥1	3	12	4	1	1	2	1	0
Cefuroxime	≤8	16	≥32	15	0	5	0	3	0	1	0
Nalidixic acid	≤8	—	≥32	0	15	4	1	1	2	1	0

S: susceptible, I: intermediate, R: resistant.

## Data Availability

The data that support the findings of this study are available from the corresponding author upon reasonable request.

## Abbreviations

CLSI: Clinical and Laboratory Standards Institute guidelines
ESR: Erythrocyte sedimentation rate
HIV: Human immunodeficiency virus
iNTS: Invasive nontyphoidal salmonella
KMCH: Kasturba Medical College Hospital
KMC: Kasturba Medical College
NTS: Nontyphoidal salmonella
WBC: White blood corpuscles.

## Additional Points

*Highlights*. (i) Invasive infections by nontyphoidal salmonella (NTS) are seen in diabetic and cancer patients. (ii) NTS isolates were resistant to multiple antibiotics. (iii) Drug-resistant NTS are a cause of concern.
